# Selenium as an Essential Micronutrient: Roles in Cell Cycle and Apoptosis

**DOI:** 10.3390/molecules14031263

**Published:** 2009-03-23

**Authors:** Huawei Zeng

**Affiliations:** United States Department of Agriculture, Agricultural Research Service, Grand Forks Human Nutrition Research Center, Grand Forks, North Dakota 58202-9034, USA; E-Mail: huawei.zeng@ars.usda.gov; Tel.: +1-7017958465; Fax: +1-7017958220.

**Keywords:** Selenium, Cell proliferation, Cell cycle, Apoptosis, Cancer.

## Abstract

Selenium is an essential trace element for humans and animals, and selenium deficiency is associated with several disease conditions such as immune impairment. In addition, selenium intakes that are greater than the recommended daily allowance (RDA) appear to protect against certain types of cancers. In humans and animals, cell proliferation and death must be regulated to maintain tissue homeostasis, and it has been well documented that numerous human diseases are directly related to the control of cell cycle progression and apoptosis. Thus, the elucidation of the mechanisms by which selenium regulates the cell cycle and apoptosis can lead to a better understanding of the nature of selenium’s essentiality and its role in disease prevention. This article reviews the status of knowledge concerning the effect of selenium on cell cycle and apoptosis.

## 1. Introduction

Selenium (Se) was discovered by Berzelius in 1817, and has been recognized as an essential trace element for animals including humans [1,2]. In 1957, it was demonstrated that trace amounts of Se protected against liver necrosis in vitamin E deficient rats and hence established its nutritional essentiality [3]. The daily requirement for Se in adults (55 µg) is met by most Americans [4], however, certain populations in Europe, Asia, and parts of Africa have intakes much less than 55 µg/d, and Se deficiency is associated with disease conditions. In parts of China, very low intakes (< 25 µg/d) may contribute to a type of juvenile cardiomyopathy (Keshan disease) that is preventable by Se supplementation [5].

It is known that Se supports the expression of a variety of selenoproteins through the tRNA-mediated incorporation of selenocysteine. These selenoproteins include glutathione peroxidases (GPX) and thioredoxin reductases (TrxR), which have important antioxidant and detoxification functions [6]. In addition, Se intakes that are greater than the RDA appear to be beneficial. In the late 1960s, it was first suggested that Se might also be anticarcinogenic, based on an inverse relationship of Se status and risks of some kinds of cancer [7,8]. Since then, a substantial body of persuasive evidence indicates that Se can, indeed, play a role in cancer prevention including epidemiological evidence, cell and animal studies, and human intervention findings [9,10,11,12,13,14]. Interest in this area was stimulated by the landmark finding that supplementation of free-living (no restriction on diets and life style) people with Se-enriched brewer’s yeast containing predominantly selenomethionine (SeMet) but also trace amounts of other Se forms (i.e. Se-methyl-selenocysteine, γ-glutamyl-Se-methylselenocysteine, Se-adenosyl selenohomocysteine) decreased the overall cancer morbidity by nearly 50% [15,16]. That finding came from the Nutritional Prevention of Cancer (NPC) Trial, a prospective, double-blinded, randomized, placebo-controlled trial involving 1,312 patients recruited because of histories of non-melanoma skin cancers, i.e., basal cell and/or squamous cell carcinomas. The recent Selenium and Vitamin E Cancer Prevention Trial (SELECT) data [17] showed that Se did not help prevent prostate cancer. This is somehow unexpected because most previous studies have demonstrated the Se’s anticancer potential [1,2,3,4,5,6,7,8,9,10,11,12,13,14,15,16]. It has been suggested that the l-SeMet given in the SELECT trial may have been less active than Se chemical forms (high-Se yeast) given in the NPC trial [17], which may have at least in part contributed to the failure of l-SeMet to prevent prostate cancer in the SELECT trial. The fact that 200 μg Se/d of l-SeMet but not high-Se yeast caused a health safety issue in the SELECT trial further suggests the significant difference of Se chemical forms between l-SeMet and high-Se yeast. This underscores the urgency of understanding Se chemical forms, doses and their molecular targets.

It has been well recognized that Se plays a key role in cell cycle and apoptosis but mechanisms for Se action are not fully understood. In humans and animals, cell proliferation and death must be regulated to maintain tissue homeostasis. The eukaryotic cell cycle is divided into four major phases as follows: the G1 phase before DNA replication, the periods of DNA synthesis (S phase), the G2 phase before cell division and cell division (M phase). The cell cycle is a conserved mechanism by which eukaryotic cells replicate themselves, and apoptosis is also a highly conserved mechanism by which eukaryotic cells commit suicide [18]. Apoptosis enables an organism to eliminate unwanted and defective cells during normal development, turnover and pathological conditions. Recent research has shown that the effectiveness of Se compounds as micronutrients and chemopreventive agents correlates with Se’s chemical form and doses. There are several proposed mechanisms to explain the effect of Se on cell cycle and apoptosis, and these mechanistic aspects may shed light on how Se acts as a micronutrient / chemopreventive agent.

## 2. Se biochemistry/metabolism

**Figure 1:**
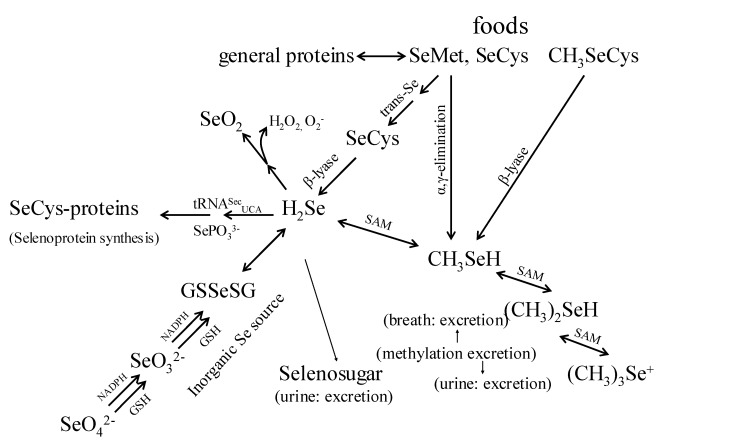
Proposed pathways for the metabolism of biologically important selenomolecules (Adapted from [12,13,14]).

Foods contain diverse amounts and chemical forms of Se, and Se is covalently bonded in multiple organic molecules. The chemical and physical properties of Se are very similar to sulfur; Se is biologically active in a variety of covalent compounds including inorganic salts, amino acids, and methylated compounds. The compounds available for use as Se-supplements include the inorganic forms, sodium selenite, sodium selenate; and the organic forms, SeMet, Se-methylselenocysteine (SeMSC), and high-Se yeast [16,19]. These Se forms are not metabolized similarly. Although the metabolism of both organic and inorganic Se-forms shows certain similarities (Figure 1), it has been found that humans absorb and retain Se better from SeMet and Se-yeast than from the inorganic Se-salts [20,21,22]. The organic forms contain Se in the reduced state (selenide: Se(-II)) while the inorganic salts contain Se in oxidized forms (selenite: Se(IV); selenate: Se(VI)). Upon absorption of these Se compounds, the higher-valence forms are reduced to the selenide state using reducing equivalents from reduced glutathione and NADPH, and the organic forms such as SeMet, selenocysteine (SeCys) release Se in the selenide state as a result of catabolism. The Se from SeMet, consumed in the form of food proteins and/or dietary supplements, is thus metabolized to form SeCys. Alternatively, SeMet can also be incorporated non-specifically into proteins, as it freely substitutes for methionine in protein synthesis. It is noted that intact SeCys from human diets is not immediately used for protein synthesis, instead, it is cleaved to form selenide (H_2_Se) [22]. 

Selenide is the branch point of two metabolic pathways. The first pathway is responsible for selenoprotein production which includes the co-translational biosynthesis of SeCys and its incorporation into specific selenoproteins. This process involves the loading of a specific tRNA with the amino acid serine by seryl-tRNA synthase, followed by the SeCys synthase-catalyzed replacement of the serinyl hydroxyl with a selenol moiety (–SeH) from selenophosphate to form tRNA-bound SeCys [23,24]. With SeCys-insertion sequence (SECIS) elements, the process is unique in that the tRNA involved is recoded such that UGA, normally a termination codon, specifies the co-translational insertion of SeCys [1, 23]. Because selenoprotein expression is tightly regulated, the second pathway is that Se in excess of these needs enters an excretory pathway, and methylation and sugar-derivation of selenides form the major excretory products (Figure 1) [24,25].

Several lines of evidence suggest that methylselenol produced in the excretory pathway is a key *in vivo* anticarcinogenic metabolite [6, 26]. When arsenic was used to block Se-methylation, it reduced the capability of metabolic precursors of methylselenol to support selenoprotein expression, but enhanced the anti-tumorigenic activities of such precursors in animal models and these studies demonstrated that Se-antitumorigenesis, depends on the metabolic production of methylselenol [27,28] (Figure 1). 

It is known that Se-enriched yeast contains SeMet and trace amounts of other Se forms [15,16]. Are there synergetic effects of SeMet and other Se forms on biological function such as cell cycle and apoptosis? How do SeMet and these trace amounts of other Se forms regulate cellular insulin signaling pathway? These future studies may further our understanding of the outcome of the SELECT results regarding the lack of anti-cancer effect of L-SeMet and an increase in diabetes.

## 3. The effect of Se on cell cycle and apoptosis at nutritional doses

Intake of Se, which ranges from clearly deficient to nutritional doses, plays a vital part in many metabolic functions. The extreme situations of deficiency and nutritional doses are relatively straightforward. Our dietary Se comes largely from consumption of bread, cereals, fish, poultry and meat. The selenoproteins are required for normal health, and the best examples are the antioxidant GPX enzymes and redox regulators in mammalian cells. These GPX enzymes eliminate hydrogen peroxide and damaging lipid and phospholipid hydroperoxides generated *in vivo* by free radicals and other oxygen derived species [29]. The importance of Se has been further demonstrated when Se responsiveness of Keshan disease, an endemic fatal cardiomyopathy, was found in areas of China with particularly low soil Se [5]. In addition, human Se deficiency was reported in patients on long-term parenteral nutrition and preterm infants experience significant Se depletion [30]. Selenium is a potent effector of cell growth with a relatively narrow window of tolerance. In the form of selenite, SeMet or SeCys, it functions as an essential micronutrient at levels of ~ 0.1-0.2 μg/g in the diets of experimental animals and livestock, but it becomes a toxic at levels exceeding 5 μg/g [31]. The recommended daily allowance for Se is 55μg/d for both men and women. Doses of 100-200 μg Se/d inhibit genetic damage and cancer development in human subjects, and about 400 μg Se/d is considered an upper safe limit [10]. At nutritional levels, Se is the defining component of the SeCys-containing selenoproteins, a family whose members exhibit a wide range of functions, including roles in cellular antioxidative protection, redox regulation, male fertility, and thyroid function [32]. Selenium has been suggested to mediate a number of insulin-like actions including the stimulation of glucose uptake and regulation of glycolysis, gluconeogenesis, fatty acid synthesis and the pentose phosphate pathway [33]. However, it was recently documented that the development of insulin resistance in mammals was associated with elevated expression of an antioxidant enzyme such as GPX1, and these data suggested that increased GPX1 activity may interfere with insulin function by overquenching intracellular reactive oxygen species required for insulin sensitizing [34,35]. This observation is consistent with the findings of an increase in self-reported diabetes in the SELECT trial [17].

It has been well documented that the antitumorigenicity of Se involves immunologically based cellular responses, and Se status can affect immune functions [36]. At human nutritional doses, Se is essential for an optimum immune response and affects both the innate and acquired immune systems. For example, Se-deficient lymphocytes are less able to proliferate in response to a mitogen, but the response can be improved by Se supplementation [37]. Although the effect of Se on the immune response may be directly related to the role of Se in cell cycle and apoptosis, the mechanisms of Se involvement for immune cell proliferation *in vivo* are not fully understood, and most data are from *in vitro* studies.

**Figure 2:**
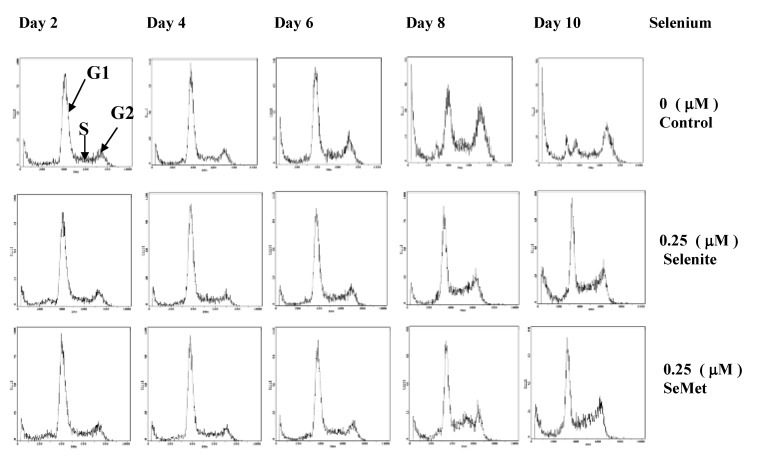
Flow cytometric profiles show cell cycle phase-distribution of the HL-60 cells in the absence of Se or in the presence of 0.25 μmol/L selenite / SeMet (Adapted from [40]).

Selenium, at nmol/L concentrations, is also essential for the growth of certain cells in culture. It has been shown that selenite at 50 nmol/L, but not at > 1 μmol/L, is necessary for growth of WI-38 diploid human fibroblasts, Chinese hamster cells and enhances growth of other human cell lines [38,39]. Our previous study suggested that when HL-60 cells (derived from human lymphocyte cells) were optimized in serum-free culture conditions to maximize the effects of Se on cell growth, Se (nmol/L) as either selenite or SeMet was an essential nutrient for cell proliferation [40]. In fact, the finding that Se enhances growth of cells in culture [38,39] is well recognized; up to 100 nmol/L selenite is added in some commercial cell culture media such as IMDM media (Invitrogen, CA) for optimal growth of certain mammalian cell lines. Our cytometric analysis [40] suggested that Se (nmol/L) either selenite or SeMet played a critical role in the dynamics of cell cycle progression, when G1, S and G2 phase distribution of cells supplemented with nmol/L selenite or SeMet was compared with that of Se-deficient cells over time. Although G1 phase cell distribution of both Se-deficient cells and the cells supplemented with selenite or SeMet decreased during up to 10 day incubation period, the decrement was more dramatic in Se deficient cells. On the other hand, G2 phase cell distribution gradually accumulated on days four and six and became significantly increased on day eight and 10 in Se-deficient cells. In addition, the apoptosis rate of Se-deficient cells appeared to be higher than that of the cells supplemented with nmol/L selenite or SeMet (Figure 2) [40]. 

The eukaryotic cell cycle progression is orchestrated by cyclin-dependent kinases (cdks) which are modulated through association with their regulatory subunits, the cyclins [41,42]. Our data suggested that both selenite and SeMet up-regulated the expression of numerous cell cycle-related genes, and these genes included c-Myc, cyclin C, proliferating cell nuclear antigen, cyclin-dependent kinase (cdk)1, cdk2, cdk4, cyclin B and cyclin D2 mRNA. In addition, Se increased total cellular phosphorylated proteins [40]. The Se-induced up-regulation of c-Myc and cyclin C mRNA levels is consistent with the observation that cyclin C can cooperate with c-Myc in the promotion of cell proliferation [43]. Cyclin/cdk1 is thought to be the major kinase that initiates the onset of mitosis, and cdk2 and cyclin B are essential for the transition of S/G2 phases, initiation of DNA synthesis, activation of cdk1 and entry into mitosis in higher eukaryotes [44]. In response to mitogenic stimuli in G1 phase, it was reported that cdk4 associated with the D-type cyclins that control cell cycle progression by phosphorylation of the tumor suppressor protein, pRb [45]. Thus, the up-regulation of cdk1, cdk2, cdk4, cyclin B and cyclin D2 led to the promotion of cell cycle progression, particularly G2/M transition and /or the reduction of apoptosis, *in vivo* and *in vitro* [40,46]. Similarly, when grown in serum-deficient medium, TrxR 1 deficient cells lose self-sufficiency of growth, manifest a defective progression in their S phase and a decreased expression of DNA polymerase α, an enzyme important in DNA replication [47]. In agreement with these data, it was found that Se deficiency caused a significant increase in reactive oxygen species (ROS), especially lipid hydroperoxides inside cells [48]. When Jurkat cells were cultured in a serum-free (Se-deficient) medium, selenoenzyme activities such as GPX and TrxR decreased significantly within cells and subsequently induced cell death [48]. Interestingly, a lipid soluble radical-scavenging antioxidant (vitamin E) but not the water-soluble antioxidants (ascorbic acid, *N*-acetylcysteine, and glutathione), completely blocked Se deficiency-induced cell death, although vitamin E could not restore Se-dependent enzyme activity [48]. Thus, these data suggest cellular ROS, especially lipid hydroperoxides, are directly involved in the cell cycle arrest and apoptosis caused by Se-deprivation. In addition, Se compounds may modulate a variety of cellular activities including cell survival and death. At nutritional doses, Se is likely to prevent the stimulation of the caspase-3-like protease activity and PARP cleavage that occurs from cell death induced by oxidant stress [49]. 

As mentioned, under normal physiological conditions, human cells cannot tolerate Se-deficiency because Se is an essential nutrient. However, most hepatocellular carcinoma cell lines (10 of 13) tolerate Se deficiency, and escape the consequent cell death induced by this [50]. Our recent data [51] also showed that Se-deficiency did not affect cell cycle progress and apoptosis in human colon Caco-2 cells even though GPX activity of Se-deficient cells was 11 mU/mg protein compared to 140 mU/mg protein in cells supplemented with Se. Our further studies demonstrate that although Caco-2 cells are resistant to Se deprivation, Se may exert its anticancer property by increasing the expression of humoral defense gene (A2M) and tumor suppressor-related genes (IGFBP3, HHIP) while decreasing the expression of pro-inflammatory genes (CXC L9, HSPB2) [51]. Thus, cell proliferation of certain cell types such as immune cells is extremely sensitive to Se-deprivation while others are not. However, Se may still modulate the gene expression in those cells which tolerate Se deficiency. 

Although Se at nutritional doses has been implicated to play a key role in the cell cycle and apoptosis in certain cultured cell types [38,39,40, 46,47,48,49,40,46], the effective plasma levels of Se are still unknown, and more *in vivo* studies are needed. In addition, more different cultured cell types, Se chemical forms and other experimental approaches such as the detection of BrdU incorporation rate and double-thymidine-block release, should be used in the future studies. These data and the characterization of Se’s effect on cellular insulin signaling pathway will further our understanding of Se’s nutritional role and its anticancer potential.

## 4. The effect of Se on cell cycle and apoptosis at supranutritional doses

A body of evidence indicates that a possible mechanism by which Se reduces the cancer risk is through its effects on cell cycle and apoptosis [52,53,54,55]. Work with a gene therapy strategy demonstrated that L-methionine-gamma-lyase (METase) expression in tumor cells converts SeMet into the Se metabolite, methylselenol which activates the caspase cascade and apoptosis in the tumor cells [54]. Furthermore, *in vivo* SeMet treatment of nude mice bearing tumor cells that express METase significantly inhibited ascites tumor growth and prolonged host survival [54]. In addition, submicromolar methyselenol caused cell cycle arrest, and led to an increase in the G1 and G2 fractions with a concomitant drop in the S-phase [55,56]. 

There are several important factors that should be considered concerning Se and cell cycle/ apoptosis. In most cases, organic Se compounds are less toxic but more potent than inorganic Se ones. It is generally believed that the anticancer effects of Se involve the perturbation of tumor cell metabolism. Both inorganic and organic Se-compounds can be anti-tumorigenic at “supranutritional” doses, but their molecular response pathways are different [57]. Early studies demonstrated that there were two types of cellular responses: (1) growth inhibition was caused by DNA single-strand breaks and genotoxic effects, which is accompanied by a decrease in cell proliferation and an increase in cell death due to necrosis and apoptosis; (2) growth inhibition was caused by specific inhibition of cell cycle regulatory protein, and apoptosis which is the key mechanism with little involvement of DNA single-strand breaks [57,58]. For example, cultured cancer cells exposed to high levels of inorganic Se (selenite) *in vitro* have been found to show predominantly non-specific genotoxic effects which are manifested by single and double strand DNA breaks, and reductions in total DNA, RNA and protein synthesis. Cell death, DNA apoptotic fragmentation, and DNA double stranded breaks were preceded by the occurrence of DNA single stranded breaks detected using a filter elution assay [59]. It is known that cell cycle progression is governed by protein kinases, the cyclin-dependent kinases (cdks), that are activated at specific cell cycle stages. Cdk2 acts at S phase and it binds to cyclins D and E in early and late G1. Cells exposed to inorganic Se (selenite) were arrested at the S/G2-M phases with an increase in cdk2 kinase activity and DNA damage-inducible gadd gene [55,56,57,58,59,60]. In addition, selenite treatment was found to increase the duration of various phases of the cell cycle, inhibiting cell growth. *In vivo* feeding of high levels of dietary selenite to rats increased concentrations of both reduced glutathione (GSH) and oxidized glutathione (GSSG) with a decrease in GSH:GSSG ratio [61]. The higher concentrations of selenite can overproduce selenodiglutathione (SDG), the initial Se metabolite, which inhibits the cell growth, and induces apoptosis [36]. These observations are consistent with the fact that chemopreventive efficacy varies among Se-compounds [11]. Hydrogen selenide (H_2_Se) and CH_3_SeH are major pools of Se metabolites that induce distinct types of biochemical and cellular responses [62,63]. The H_2_Se precursors, selenite and SeCys induced DNA single strand breaks (genotoxicity) [64,65,66,67], and selenite at 5-10 μmol/L caused extensive cytoplasmic vacuolization of cells, cell detachment and cell membrane leakage. The fact that a superoxide dismutase (SOD)-mimetic (copper dipropylsalicylate/Cu^++^) blocked DNA single strand breaks and apoptosis [64,68] suggested that these effects directly involved redox-mediated effects of selenite. Subsequently, the role of superoxide generation by the H_2_Se pool was confirmed using SOD or SOD-mimetics [69,70], and the generation of ROS was detected in *in vitro* models by the reaction of selenite with GSH and other thiol compounds [71,72]. Consistent with the above findings, selenite (a precursor of H_2_Se) exposure caused S-phase cell arrest, induced DNA single strand breaks, and caused ROS-induced p53 activation leading to superoxide/p53/Bax-mediated activation of mitochondrial pathway. In addition, selenite mediated caspase-independent apoptotic DNA fragmentation with decreased expression of p27kip1 and p21cip1 and increased phosphorylation of AKT, JNK1/2, and p38MAPK [69,73,74]. Several studies have also demonstrated selenite-induced apoptotic DNA laddering in the p53-mutant cancer cells without the cleavage of poly(ADP-ribose) polymerase (PARP; i.e., caspase-independent apoptosis); whereas CH_3_SeH and its metabolic precursors induced caspase-mediated apoptosis in those cells [75,76,77]. In p53 wild type cancer cells, selenite also activated the caspase-mediated apoptosis involving both the caspase-8 and the caspase-9 pathways [78]. Further studies indicated that selenite induced a rapid superoxide burst and p53 activation, leading to Bax up-regulation and translocation into mitochondria, which restored the cross-talk with stalled tumor necrosis factor-related apoptosis-inducing ligand (TRAIL) signaling for a synergistic caspase-9/3 cascade-mediated apoptosis execution [79].

In contrast, metabolic precursors of CH_3_SeH (methylselenocyanate, SeMSC) exerted moderate anti-proliferative effects as assessed by ^3^H-thymidine incorporation into DNA of the cells at the G1 phase of the cell cycle, whereas selenite rapidly blocked DNA synthesis and arrested cells in the S phase [55,56,80]. Cells exposed to organic Se (MSC; methylselenocyanate, MSeCN) were arrested at the G1 phase of the cell cycle as there was a decrease in the cdk2 kinase activity accompanied by a decrease in cyclin E-cdk2 content [57]. Similarly, exposure of DU145 human prostate cancer cells to methylseleninic acid (MSeA), a CH_3_SeH precursor, led to a profound G1 arrest and increases in Bax/Bcl-2 ratio, DNA fragmentation and caspase-mediated cleavage of PARP. Apoptosis in the prostate cancer cells was accompanied by dose-dependent decreases in phosphorylation of protein kinase AKT and extracellular signal-regulated kinase (ERK1/2) but the phosphorylation of p38 mitogen-activated protein kinase (p38MAPK) and c-Jun NH_2_-terminal kinase (JNK1/2) was unchanged. Although p53 activation is not absolutely required, phosphorylated p53 plays a critical role in CH_3_SeH-induced apoptosis [81, 82]. More recent data showed that MSeA up-regulated cdk inhibitors (cdkis) such as P16/INK4a, P21/CIP1 and P27/KIP1. These cdkis bind with their respective cylin/cdk complexs and inhibit the kinase activities of cdk4, cdk6 and cdk2. Consequently, it caused G1 cell cycle arrest in vascular endothelial cells [83]. In contrast, previous data demonstrated that MSeA induced G1 cell cycle arrest through a reduction of total cellular level of cyclin D1 and cdk4 protein [80]. Although methylselenol induces the same G1 cell cycle arrest, most findings on methylselenol-related molecular targets are likely cell or tissue type dependent, and it involves different cyclin/cdk/cdki and upstream mitogenic signaling pathways. 

Selenium also plays several other key roles in anticancer cellular signaling and gene transcription, and biochemical reactions of Se with protein thiols have been documented [26]. Protein kinase C (PKC) represents a family of phospholipid-dependent serine/threonine kinases that are involved in a variety of pathways that regulate cell growth, death and stress responsiveness [84,85]. Thus, PKC is a logical molecular target for redox modification by Se compounds that may in part determine their anticancer activities. Because of the high reactivity of anionic CH_3_Se-, it may disrupt disulfide bonds in cysteine-rich regions of proteins. The redox-mediated inactivation of PKC may be responsible, at least in part, for the antioxidant-induced inhibition of tumor promotion and cell growth, as well as for the induction of cell death [86]. Methylselenocysteine and MSeA, which can be generated locally by the reaction of membrane CH_3_SeH with PKC-bound tumor-promoting fatty acid hydroperoxides, selectively inactivates PKC [86, 87]. More recent data indicated that MSeA-induced growth inhibition and apoptosis decreased with a conditional overexpression of PKCε and increased with its knock-out by small interfering RNA [87]. These data suggest that when MSeA is generated within the vicinity of PKC, it specifically inactivates PKC isoenzymes, which causes growth inhibition and apoptosis [88]. Several studies also showed inhibitory effects of MSeA on other protein kinase pathways including phosphatidylinositol 3-kinase (PI3K) and phospho-extracellular signal regulated kinase (ERK) 1/2 andphospho-JNK [89,90]. Activation of p38 MAPK may also be involvedin vascular endothelial apoptotic responses in a pharmacologicalor therapeutic context of MSeA exposure [89,90]. 

The results of microarray profiling analyses have indicated that Se-treatment can alter several genes related to cell cycle/apoptosis in a manner related to cancer prevention [91]. Treatment with Se resulted in the up-regulation of genes involved in phase II detoxication enzymes, in certain Se-binding proteins, and in some apoptotic genes [91]. Selenium treatment also resulted in the down-regulation of genes related to phase I activating enzymes and cell proliferation [91]. In all tissues tested, Se-treatment arrested cells in the G1 phase of cell cycle, inhibited the expression of the cyclin A, cyclin D1, CDC25A, CDK4, PCNA and E2F genes, while inducing the expressions of P19, P21, P53, GST, SOD, NQO1, GADD153 and certain caspase genes [91]. In human breast cells, Se affects genes falling into three categories: cell cycle checkpoint controllers (e.g., cyclins, cdcs, cdks, E2F family proteins, and serine/threonine kinases), apoptosis regulatory genes (e.g., Apo-3, c-jun, and cdk5/cyclin D1), and signaling molecules [e.g., mitogen-activated protein (MAP)/extracellular signal-regulated protein kinase (ERK) and phosphatidylinositol 3’-kinase (PI3k) cascade genes] [92]. More recent data further suggest that Se induced prostate cancer cell apoptosis and growth inhibition through the interplay of AKT, AR, and TGFβ signaling pathways [93].

## 5. Conclusions

**Figure 3:**
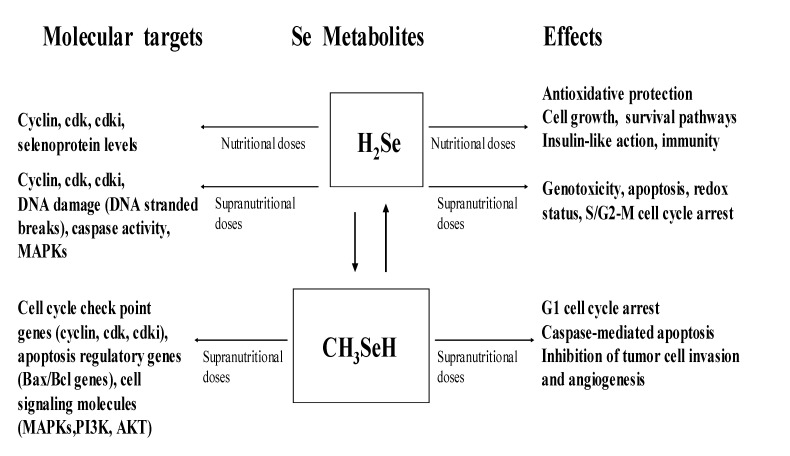
molecular targets and cellular effects of hydrogen selenide and methylselenol (Adapted from [12,63]).

Selenium is an important micronutrient, and the dominant forms of Se found in foods are SeMet and SeCys, with smaller amounts of methylated selenides. Dietary supplements may include inorganic Se-salts such as selenite and selenate. At nutritional doses, Se is an essential component of SeCys in selenoproteins, and it promotes cell cycle progression and prevents cell death. In contrast, at supranutritional doses that are greater than the nutritional requirement but not toxic, Se induces cell cycle arrest and apoptosis. The Se-modulation of cell cycle and apoptosis is a key mechanism by which Se exerts its biological functions. These include anticarcinogenic activity, protection against oxidant damage or aging, and even a role in reproduction and detoxicity. Importantly, the molecular targets and the effectiveness of Se’s action are dictated by its chemical forms and doses (Figure 3). 

## Abbreviations

AR: androgen receptor
CDK: cyclin-dependent kinase
CDKI: cyclin-dependent kinase inhibitor
ERK: extracellular signal regulated kinase
GPX: glutathione peroxidase
JNK: c-Jun N-terminal Kinase
MAPK: mitogen-activated protein kinase
MSeA: methylseleninic acid
NPC: Nutritional Prevention of Cancer
PI3K: phosphatidylinositol 3-kinase
PKC: protein kinase C
ROS: reactive oxygen species
SELECT: Se and Vitamin E Chemoprevention Trial
Se: selenium
SeCys: selenocysteine
SeMet: selenomethionine
SeMSC: Se-methylselenocysteine
SOD: superoxide dismutase
TGFβ: transforming growth factor β
TRAIL: tumor necrosis factor-related apoptosis-inducing ligand
TrxR: thioredoxin reductase
